# Medical Student Assessment of Pediatric Patient Pain as a Function of Perceived Child Gender

**DOI:** 10.1001/jamanetworkopen.2021.13010

**Published:** 2021-06-09

**Authors:** Alexander W. T. Reardon, Brian D. Earp, Patricia Andreski, Joshua T. Monrad, Lindsey L. Cohen, Gary L. Freed

**Affiliations:** 1 University of Michigan Medical School, Ann Arbor; 2 Yale University, New Haven, Connecticut; 3 Department of Psychology, Georgia State University, Atlanta

## Abstract

This survey study investigates whether the perceived gender of a child is associated with how medical students assess a pediatric patient’s pain displayed and pain experienced.

## Introduction

In 2 prior studies,^1,2^ individuals were shown a video of a 5-year-old child undergoing blood collection. When told the child was a boy, they rated the child as experiencing more severe pain.^1,2^ This study sought to assess the association of perceived child gender with medical students' assessment of the level of pain the child was experiencing.

## Methods

The University of Michigan institutional review board deemed this survey study exempt from review because it involved benign behavioral interventions. This study followed the Strengthening the Reporting of Observational Studies in Epidemiology (STROBE) reporting guideline.

In September 2019, all students at 3 Michigan medical schools were invited via email to view the same video used in the previous studies^1,2^ and to complete an online survey (participants received a $5 incentive). The child in the video had given assent and parental permission was given orally. Students were randomly told the child—previously validated as gender neutral in apperance^2^—was a “boy” or “girl.” Participants were excluded for incorrectly defining the primary response measures of pain sensation and pain display, or, after viewing the video, incorrectly identifying the described gender (boy or girl) or hair color of the child. We asked participants: “How much pain did [he/she] *experience* and *display* during the finger stick?” We recorded ratings on 2 visual analog scales ranging from 0 (no pain) to 100 (severe pain). Subsequent questions addressed explicitly held gender stereotypes, stage of medical training, completion of pediatrics clerkship, and career interest in pediatrics.

A priori analysis indicated 550 responses were needed to detect between-group differences with 1-tailed mean testing (*t* and analysis of variance) with cutoffs of α = .05 and β = 0.20 and effect size Cohen *d* = 0.21.^2^ Statistical analysis was performed using SAS statistical software version 9.4 (SAS Institute) from December 2019 to January 2020.

## Results

Among 2712 invited medical students, 702 (25.8%) responded; 203 were excluded for failing the attention or comprehension verifications. Among the 499 medical students included in the study, 326 [65.3%] were female, 173 [34.7%] were male, and 147 [29.5%] completed a pediatrics clerkship; 248 were randomly assigned to the boy condition and 251 to the girl condition (Table).

**Table.** Characteristics of Participants

Characteristic	Participants, No. (%) (N = 499)	
	Condition	
	Told boy (n = 248)	Told girl (n = 251)
Participant gender		
Male	85 (34.3)	88 (35.1)
Female	163 (65.7)	163 (64.9)
Time in medical school		
Year 1	79 (31.9)	82 (32.7)
Year 2	66 (26.6)	70 (27.9)
Year 3	56 (22.6)	52 (20.7)
Year 4	42 (16.9)	45 (17.9)
Dual degree	3 (1.2)	2 (0.8)
Pediatrics clerkship		
Complete	77 (31.0)	70 (27.9)
Incomplete	169 (68.1)	181 (72.1)

The mean (SD) experienced pain ratings did not significantly differ between those assigned to the boy condition (48.7 [18.3]) vs the girl condition (46.9 [19.3]) condition, nor did they significantly differ between male (48.6 [19.3]) and female (47.3 [18.6]) medical students, regardless of assigned condition.

There was, however, a significant difference between mean ratings, for all participants, of the child’s experienced pain vs displayed pain (mean [SD] experienced pain: 47.8 [18.8]; mean [SD] displayed pain: 68.5 [17.4]; difference: 20.7 [95% CI, 18.4-23.0]; *P* < .001). Students having completed a core pediatrics clerkship (n = 147) rated experienced pain as less severe than did students with less pediatric clinical experience (mean [SD] pain experienced rating by students who had completed a pediatric clerkship: 42.6 [19.0] vs those who had not: 50.0 [18.3]; difference: 7.4 [95% CI, 3.8-11.0]; *P* < .001) (Figure). There was no significant difference between these 2 groups’ ratings of displayed pain.

**Figure.**
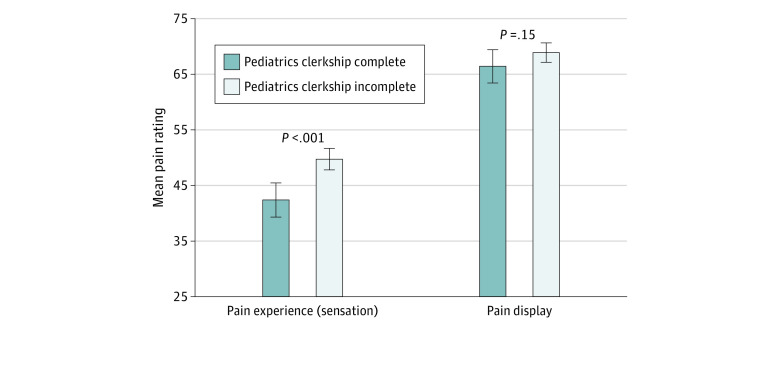
Comparing Mean Pain Experience and Pain Display for Preclinical and Clinical Students Error bars denote 95% CIs.

## Discussion

Previous studies using this video showed that among US undergraduate psychology or nursing students^1^ and a general-population sample,^2^ the experienced pain of a child was judged significantly differently if described as a boy vs girl. This study of medical students, by contrast, did not. It is unknown whether this null finding reflects gender-focused efforts in medical education, preexisting differences in attitudes among the samples, or insufficient power to detect an association.

More experienced medical students (ie, having completed their pediatric clerkship) evinced a widened gap between their assessments of pain severity displayed vs experienced by the child. This gap might be interpreted as an impression of the child overreacting to a painful stimulus. Hence, our results may signal an association between greater pediatric clinical experience and an increased tendency to discount (or become inured to) the degree of pain displayed by a child when inferring the degree experienced.

Study limitations included failure to collect information on potential mechanisms (eg, number of blood draws witnessed or performed). It is also unclear whether the findings are clinically significant given that, for example, these participants were students and not practicing professionals. Further study is required of the potential impact of different medical education experiences on students’ assessments of patient pain.
